# Therapeutic potential of mesenchymal stem cell-derived exosomes and miRNAs in neuronal regeneration and rejuvenation in neurological disorders: a mini review

**DOI:** 10.3389/fncel.2024.1427525

**Published:** 2024-10-04

**Authors:** Aria Salehpour, Zahra Karimi, Mokhtar Ghasemi Zadeh, Mohammadreza Afshar, Ali Kameli, Fatemeh Mooseli, Masoud Zare, Alireza Afshar

**Affiliations:** ^1^The Persian Gulf Marine Biotechnology Research Center, The Persian Gulf Biomedical Sciences Research Institute, Bushehr University of Medical Sciences, Bushehr, Iran; ^2^Student Research Committee, Bushehr University of Medical Sciences, Bushehr, Iran

**Keywords:** neurological disorders, exosomes, mesenchymal stem cells, MSCs, regenerative medicine

## Abstract

Mesenchymal stem cells (MSCs) have gained considerable attention in the field of regenerative medicine due to their ability to secrete small extracellular vesicles (EVs) known as exosomes. This review delves into the various biological activities of MSCs and the cell interactions enabled by these exosomes, with a focus on their potential for neuronal regeneration and the treatment of neurological disorders. We scrutinize findings from multiple studies that underscore the neuroprotective and neuro-regenerative effects of exosomes derived from MSCs, illuminating their mechanisms of action and therapeutic applications. This review thoroughly investigates all related pathways, miRNAs, and factors to suggest potential strategies for enhancing therapy for neurological disorders using exosomes and miRNAs, and for boosting neuronal regeneration.

## 1 Background

Neurological illnesses accounted for the second highest cause of mortality and the largest cause of disability in 2016 (Feigin and Vos, 2019). Globally, around 30% of individuals may have a neurological problem at some point in their lives (Feigin and Vos, 2019). Stroke, migraine, Alzheimer’s disease, and other dementias are the most prevalent neurological reasons for disabilities and the number of fatalities has climbed by 39% and the number of life years lost due to disability has decreased by 15% in the last 30 years (Feigin and Vos, 2019; Feigin et al., 2020).

There has been significant progress in understanding the mechanisms of nerve regeneration (Wareham et al., 2022). MSCs have emerged as a promising treatment for nerve injury due to their ability to differentiate into various cell types and secrete a range of bioactive molecules (Xia et al., 2023). The colony-forming fibroblastic cells were extracted from the bone marrow by Friedenstein and colleagues, who were the first to study their properties (Friedenstein et al., 1968).

These cells were distinguished by their preferential adhesion to tissue culture plastics. The groundbreaking research of Friedenstein et al. (1968) was expanded upon by a number of other groups, who demonstrated that these malleable adherent human cells produced from bone marrow could develop into several mesenchymal cell types, such as osteoblasts, chondrocytes, and adipocytes (Ashton et al., 1980; Bab et al., 1986; Castro-Malaspina et al., 1980). Because of their strong capacity for self-renewal and their capacity to generate bone and cartilage, these cells were dubbed “mesenchymal stem cells (MSCs)” and it was hypothesized that they were in charge of the regular turnover and upkeep of adult mesenchymal tissues (Caplan, 2005; Ma, 2010).

Most human cells excrete EVs, which transfer information from one cell to another, resulting in either positive or negative regulation (Thery et al., 2018). One subtype of EVs, exosomes, are released by eukaryotic cells into the extracellular environment and range in size from 30 to 150 nm (Li et al., 2023; Yanez-Mo et al., 2015). Exosomes are small membrane-bound vesicles that can transport and deliver proteins, lipids, nucleic acids, and other cargo to recipient cells (Gurung et al., 2021). Recent research has shown the presence of a variety of nucleic acids, including as mRNAs, miRNAs, and other non-coding RNAs (ncRNAs), in the exosomes. When exosomes circulate, these exosomal RNAs might be absorbed by nearby or distant cells, whereupon they modify recipient cells (Sato-Kuwabara et al., 2015). Exosomes also contain several cellular markers, including CD63, CD81, and CD9, which are often derived from endosomes (Kowal et al., 2016).

Exosomes produced by Mesenchymal Stem Cells (MSC-EXOs) play a crucial role in mediating cell interactions by transferring protein and DNA contents (Xin et al., 2013). Published research suggests that MSC-EXOs may promote neurogenesis, reduce neuronal cell apoptosis, stimulate the formation of new blood vessels, and alleviate local inflammation (Guo et al., 2019; Harrell et al., 2021; Qiu et al., 2015). As vital mediators of intracellular and intercellular communication, exosomes are immune-friendly and biocompatible, making them ideal native nanocarriers for stem cell (SC)-free therapy (He et al., 2018; Liu et al., 2022).

Many studies focus on methods that could support regeneration and lead to medical breakthroughs, such as enhancing neuron survival, increasing the rate of neurite outgrowth, and improving the extensive growth capacity of axons to overcome inhibitory signals. These studies aim to develop new therapies that support complete nerve regeneration (Scheib and Hoke, 2013; Uz et al., 2018). However, successful functional recovery remains insufficient and unsatisfactory (Liu et al., 2022). This situation has led to the exploration of SC-based treatments and their secretomes, such as exosomes, as alternative therapies with fewer drawbacks. This review compiles related studies to provide an overview of recent advancements and suggest potential directions for future research.

## 2 Materials and methods

The search query “(“Exosomes”[Mesh]) AND (“Neurons”[Mesh]) AND (“Mesenchymal Stem Cells”[Mesh]) “was used to gather data for this review from the Pubmed database in January 2024. The following criteria were applied to include all relevant studies:

1. Studies that cite MSCs as the source of the exosomes being used.
2. Studies that provide methods for characterizing or isolating exosomes, rather than just stating EVs or media for culturing SCs.
3. Studies that define a neurological condition that MSC-EXOs aim to target.

This review excluded other publications that either did not mention the term MSCs or did not provide identification evidence for MSCs despite claiming a similar status (such as urine-derived SCs or adipose-derived SCs).

## 3 Literature review

### 3.1 MSC-derived exosomes in various neurological disorders

MSC-EXOs have a wide range of therapeutic applications for the treatment of neurological disorders. Exosomes have been found to be beneficial against neurological disorders caused by oxidative stress, peripheral nerve injury, spinal cord injury (SCI), optic nerve damage, Parkinson’s disease, neuroinflammation, status epilepticus (SE), and hearing loss (Guy and Offen, 2020; Riazifar et al., 2019). This section offers a comprehensive review of the currently available literature on the impact of MSC-EXOs on different neurological disorders.

#### 3.1.1 Peripheral nerve injury

The crucial role of Schwann cells in healing peripheral nerve injury is underscored, as they can absorb and utilize exosomes from Umbilical Cord MSCs. A significant increase in the expression of endogenous Zeb2, c-JUN, and ERK1/2 in Schwann cells was shown after they absorbed exosomes from hypoxia-pretreated Umbilical Cord MSCs, suggesting a promising approach for nerve repair. The dosage of utilized hypoxic the exosomes was 2.76 mg/g of body weight. Local subcutaneous injection was employed to deliver the exosomes. This activation enhanced nerve regeneration and myelin repair, highlighting the potential therapeutic impact of exosomes on neural tissue (Zhu et al., 2022).

Other studies have investigated the interesting feature of fluorescent tracer uptake in MSC-EXOs and their role in supporting peripheral nerve injury (PNI) healing. MSC-EXOs have shown potential in promoting nerve regeneration and functional recovery following PNI as these exosomes mediate various biological activities and cellular interactions, including the transfer of proteins and DNA contents between donor and recipient cells (Sowa et al., 2016). Gelatin hydrogel tubes were used to implant Schwann cells and adipose-derived SCs into the mice’s artificially attenuated sciatic nerve injury model. Prior to transplantation, the gelatin hydrogel tube lumen was filled with Schwann cells and SCs generated from adipose tissue at a final density of around 1 × 10^4^ cells/tube (Sowa et al., 2016). Notably, exosomes from different sources, such as adipose tissue and bone marrow, have shown effectiveness in supporting PNI healing (Kourembanas, 2015; Liu et al., 2020).

Reactive oxygen species (ROS) increase during intervertebral disc degeneration (IVDD) due to decreased Nrf2 expression, a key transcription factor that buffers against high ROS. It was found that BMSC-EXOs reduced ROS production while also reducing the apoptosis, inflammation, and degeneration of nucleus pulposus (NP) cells by inhibiting Keap1 and promoting Nrf2 expression (Xu et al., 2023). Nuclear translocation and Nrf2 were enhanced by BMSC-EXOs, while NF-κB expression was suppressed. Antioxidative protein expression also increased after BMSC-EXOs therapy. For the first time, it was shown that BMSC-EXOs could reactivate the suppressed antioxidant response mechanisms in degenerating NP cells by modifying the Keap1/Nrf2 axis. BMSC-EXOs may be used as an immediate ROS modulator to treat intervertebral disc degeneration (Xu et al., 2023).

#### 3.1.2 Spinal cord injury

Phosphatase and tensin homolog (PTEN) protein expression in the damaged spinal cord area might be reduced by MSC-EXOs loaded with PTEN small interfering RNA (ExoPTEN) after intranasal treatments. Intranasally administered ExoPTEN resulted in significant improvements in motor function, faster recovery of the urine reflex, and sensory recovery. Physiological changes such as reduced neuroinflammation and gliosis, enhanced angiogenesis and axonal regeneration, and improvements in structure and electrophysiology were observed along with functional recovery. The practical use of this fast, noninvasive, cell-free, lesion-specific, and efficient therapy in SCI and other conditions shows considerable potential (Guo et al., 2019).

MSC-EXOs may enhance functional recovery in SCI rats by reducing neuron loss. The miR-21/PTEN/PDCD4 signaling pathway emerged as a key regulator, promoting cell viability and suppressing cell death *in vivo* (Kang et al., 2019).

Bone marrow MSC-EXO could activate the Wnt/*β*-catenin signaling pathway, which can be a potentially effective treatment for SCI. Bcl-2 protein expression increased while Bax, cleaved caspase-3, and cleaved caspase-9 protein expression decreased in response to MSC-EXOs intervention (Li et al., 2019).

MSC-EXOs modified with miR-133b may significantly aid in the recovery of neurological function in rats with spinal cord injuries by influencing the signaling pathway associated with axon recovery and the expressions of neurofilament, GAP43, GFAP, and myelin basic protein (Ren et al., 2019).

Administration of MSC-EXOs effectively can reduce inflammation after traumatic spinal cord injury (SCI) and inhibited the activation of A1 neurotoxic reactive astrocytes. Treatment with bone marrow MSC-EXOs reduced neuronal cell death, inhibited nitric oxide emission in microglia, and significantly increased human umbilical vein endothelial cell proliferation, migration, and angiogenic tubule development *in vivo* (Liu et al., 2019).

Moreover, the mechanical and thermal hypersensitivities of rats’ right hindpaw caused by nerve ligation were reversed by a single intrathecal injection of exosomes. Treatment with MSC-EXOs inhibited the increase of c-Fos, CNPase, GFAP, and Iba1 induced by nerve ligation. Evidence suggests the potential involvement of exosomes’ activities on glial and neuronal cells in their analgesic effects. Exosomes have also demonstrated anti-inflammatory and pro-neurotrophic properties in the ipsilateral L5/6 dorsal root ganglia of nerve-ligated rats by inhibiting the levels of TNF-*α* and interleukin-1β (IL-1β) and increasing the levels of IL-10, brain-derived neurotrophic factor, and glial cell line-derived neurotrophic factor (Shiue et al., 2019).

#### 3.1.3 Hypoxic neuron injuries and exosome-mediated neuroprotection

Hypoxic conditions play a crucial role in determining the impact of exosomes, particularly in hypoxic-related inflammatory conditions. Differentiated MSCs and exosomes derived from immune cells show enhanced treatment efficacy in hypoxic-related disorders. MSC exosomes can enable miR-133b translocation to astrocytes and neurons, which controls gene expression and promotes neurite rebuilding and neurological rehabilitation after stroke *in vivo* (Xin et al., 2013).

Additionally, improved axonal elongation and myelination following a stroke may be facilitated by the miR-17-92 cluster-enriched MSC-EXOs. This improved axon-myelin reconstruction could be partially mediated by the activation of the PI3K/Akt/mTOR pathway, which is initiated by PTEN inhibition (Xin et al., 2021). Also, Exosomes loaded with miR-29b-3p have demonstrated a reduction in ischemic brain damage by inhibiting neuronal death and promoting angiogenesis through the Akt signaling cascade (Liu et al., 2020).

Increased axonal elongation and myelination following a stroke may also be enhanced by miR-17-92 cluster-enriched MSC-EXOs. This enhanced axon-myelin reconstruction may be partially mediated by the triggering of the PI3K/Akt/mTOR pathway, which is initiated by a reduction of PTEN (Kourembanas, 2015). MSC-EXOs with increased miR-17-92 cluster improved axonal development via PTEN/mTOR signaling in the neurons. These findings suggest that exosomes produced by MSCs interact with cortical neurons to promote axonal development through the conveyance of biological materials, specifically miRNAs (Zhang et al., 2017).

Moreover, it was shown that both Normoxic Bone marrow MSC-EXOs and hypoxic Bone marrow MSC-EXOs have strong neuroprotective properties against pyroptosis mediated by the NLRP3 inflammasome *in vitro*. Patients may benefit from using hypoxic bone marrow MSC-EXOs to slow the progression of cerebral ischemia and hypoxia damage because they have a more noticeable protective impact than normoxic bone marrow MSC-EXOs (Kang et al., 2021).

#### 3.1.4 Neuroinflammation

Exosomes derived from lipopolysaccharide (LPS)-preconditioned bone marrow MSCs could facilitate the polarization of macrophages toward an M2 phenotype by inhibiting TSG-6. This, in turn, shuts down the NF-ΚB/NLRP3 regulating axis and expedited functional recovery in a rat model. These exosomes transformed the pro-inflammation macrophage into a pro-regeneration macrophage. Significant amounts of TNF-stimulated gene-6 (TSG-6) were found in extracted exosomes, which inhibited NF-KB and NOD-like receptor protein 3 (NLRP3) and may provide a healing strategy for peripheral nerve damage (Li et al., 2022).

Human amniotic fluid MSC-EXOs could reduce an inflammatory injury induced by an LPS-conditioned medium from microglia *in vitro*. This may have been accomplished by reducing iNOS activity and releasing resolving factors. Additionally, these vesicles may have a neuroprotective function by preventing the detrimental effects of microglial activation (Zavatti et al., 2022).

#### 3.1.5 Amyotrophic lateral sclerosis

Amyotrophic lateral sclerosis (ALS) is a rapidly progressing neurodegenerative disease characterized by the death of motor neurons in the brain and spinal cord. This eventually results in paralysis, muscular atrophy, and weakening. Despite considerable advancements in non-pharmacological therapies and pharmacological symptomatic treatments, effective disease-modifying medications are still elusive.

Exosomes derived from other SC types have the potential to mitigate oxidative damage, reinstate mitochondrial activities, restore the damaged cell endothelium, and enhance motor function in animals modeled with ALS (Wang et al., 2021). For instance, exosomes derived from adipose-derived SCs have been shown to exert a neuroprotective role on NSC-34 cells overexpressing ALS mutations *in vitro* (Bonafede et al., 2016). Recently, researchers discovered that exosomes formed from MSCs might promote tissue regeneration and lower inflammation *in vitro*; moreover, these cell-free products may protect degenerating motor neurons and provide a promising ALS treatment strategy (Gschwendtberger et al., 2023).

#### 3.1.6 Optic nerve damage

MSC-EXOs, which rely on certain miRNAs to control mTORC1 signaling, are a feasible and promising regenerative treatment for optic nerve damage. The delivery of miR-222-3p and miR-22-3p was shown to explain the regenerative effect of MSC-EXOs on axons *in vivo* (Sang et al., 2023).

It was demonstrated that treating optic nerve crush (ONC) rats with intravitreal MSC-EXOs significantly increased the longevity of retinal ganglion cells (RGCs). TNF-*α*, IL-1β, IL-6, IL-8, and MCP-1 were among the pro-inflammatory cytokines that showed a decrease in their levels, while IL-10, an anti-inflammatory factor, showed an increase. Furthermore, the injection of MSC-EXOs reduced the amount of apoptosis caused by ONC by upregulating the Bcl-2/Bax ratio and downregulating caspase-3 activity. Moreover, MSC-EXOs markedly increased AKT phosphorylation, while LY294002 reversed the effects of MSC-EXOs on apoptosis prevention. These findings showed that administering MSC-EXOs intravitreally can lessen the damage caused by ONC in a rat model (Cui et al., 2021).

#### 3.1.7 Parkinson’s disease

After Alzheimer’s disease, Parkinson’s disease (PD) is the second most prevalent neurological illness. The pathophysiologic abnormalities in the subsequent basal ganglia’s circuitry are caused by the degradation of dopaminergic neurons in the substantia nigra of the midbrain, which is the hallmark of PD (Kalia and Lang, 2016).

In a progressive PD mouse model (*α*-synuclein A53T transgenic mice), extracted exosomes during the development of dopaminergic neurons from Bone Marrow Stem Cells (BMSCs) could significantly enhance motor, learning, and memory functions. The mechanism behind this effect may be linked to altered phospholipid composition and cholesterol metabolism in hippocampal neurons (Xu et al., 2022). Another study indicated that the injection of Bone Marrow MSC-EXOs into the striatum of PD model rats resulted in a downregulation of the protein levels of IL-6, IL-1β, TNF-*α*, and ROS in the substantia nigra (Li et al., 2022).

#### 3.1.8 Status epilepticus

Status Epilepticus (SE) is a subset of epilepsy, representing a debilitating neurological disorder often associated with significant mortality and morbidity numbers. It is a life-threatening neurologic condition that occurs when a person has a continuous seizure or multiple seizures without enough time to recover between them. The incidences of SE are around 50 patients per 100,000 population per year with a mortality rate of around 2.5% (Al-Sofyani, 2021).

IL-1β-treated MSC-EXOs could prevent astrocytes and status epilepticus mice from experiencing LPS-induced inflammatory reactions, and its effects are mostly facilitated by the Nrf-2 signaling cascade. LPS-induced astrocyte inflammatory responses and astrogliosis could be strongly suppressed by the treatment of these exosomes as well (Liu et al., 2021).

#### 3.1.9 Oxidative stress related neurological disorders and MSC-EXOs

Adipose-derived MSC-EXOs may activate the Nrf2-ARE signaling pathway in hippocampus neurons, therefore suppressing oxidative stress generated by methotrexate (MTX). Furthermore, the Nrf2-ARE signaling pathway has a direct role in mitigating the hippocampal neuronal damage caused by MTX. All things considered, adipose-derived MSC-EXOs seem to have potential as medicines for treating neuronal damage caused by MTX while treating cancer (Huang et al., 2022). Additionally, exosomes derived from IL-1 MSCs conditioned may largely act through the Nrf-2 signaling pathway to suppress LPS-induced inflammatory responses in astrocytes and SE mice (Liu et al., 2021).

#### 3.1.10 Hearing loss

Umbilical cord MSC-EXOs may reveal protective benefits in the treatment of ototoxicity-induced hearing loss. Umbilical cord MSC-EXOs could restore hearing loss caused by cisplatin-induced deafness. This was linked to increased expression of glia-derived nexin, mmu-miR-125a-5p, mmu-miR-125b-5p, and mmu-miR127-5p in inner ear tissues and increasing particular growth factors, galectin-3, and fibronectin. These findings may help decreasing inflammation or protecting cochlear hair cells from harm (Tsai et al., 2021).

## 4 Strategies for improving MSC-EXOs effects

To enhance the therapeutic effects of MSC-EXOs, specific strategies as suggested by available studies can be utilized:

### 4.1 Considering miRNA role in neural regeneration

One avenue involves investigating the miRNA cargo of exosomes, as certain miRNAs have been associated with angiogenesis, neuroprotection, and neuroimmune regulation. Differentially expressed miRNAs, such as miRNA-199b-5p and miRNA-132-3p, have been identified in exosomes and may play a crucial role in boosting the neural regeneration capacity of exosome therapy (Table 1) (Liu et al., 2022).

**Table 1:** Mesenchymal stem cells’ exosomal miRNA targets and effects.

Type of cells	Disease	miRNA	Target/possible effect(s)	Reference(s)
Human umbilical cord mesenchymal/stromal stem cells (MSCs)	Retinitis pigmentosa	miRNA-21-5p miRNA-122-5p miRNA-146a-5p miRNA -let7a-5p miRNA -148a-3p miRNA -let7i-5p miRNA −222-3p miRNA -320a-3p miRNA −100-5p miRNA -26a-5p	Anti-inflammation	Zhang et al. (2022)
Bone marrow MSCs	Spinal cord injury	miR-124	Neuronal differentiation	Zou et al. (2014)
	Optic Nerve Crush Injury	miR-17-5p miR-30c-2 miR-92a miR-92a miR-292 miR-182	Neuroprotective	Mead et al. (2020)
	Hypoxia	miR-210	Increase migration of neural precursor cells	Wang et al. (2020)
	Spinal cord injury	miRNA-29b	Neuronal regeneration	Yu et al. (2019)
	Loss of retinal ganglion cells	SiRNA Argonaute 2	Neuroprotective and neuritogenic effects	Mead and Tomarev (2017)
	Recurrent laryngeal nerve injury	miR-21	Regeneration	Wu et al. (2017)
	Stroke	miR-1906	Neuroprotection	Haupt et al. (2021)
	Traumatic brain injury	miR-216a-5p	Promote neurogenesis and inhibit apoptosis	Xu et al. (2020)
	Spinal cord injury	miR-125a	Neuroprotective effect	Chang et al. (2021)
	Cognitive and behavioral distortion	miR-30c-5p miR-29b-3p miR-16-5p miR-221-3p miR-181a-5p miR-145-5p miR-31-5p miR-34a-5p miR-30c-5p miR-29b-3p miR-16-5p miR-221-3p miR-181a-5p miR-145-5p miR-31-5p miR-34a-5p miR-125b-5p miR-24-3p miR-199a-3p	Anti-inflammatory Reduce apoptotic state, increase astrocyte, and microglia activation	El-Derany and Noureldein (2021)
Wharton’s Jelly MSCs	Hypoxic–ischemic	let-7b-5p let-7c-5p let-7d-5p let-7e-5p let-7i-5p miR-9-3p miR-20a-5p miR-24-3p miR-26b-5p miR-28-5p miR-29a-3p miR-29b-3p miR-29c-3p miR-30d-5p miR-34a-5p miR-92a-3p miR-93-5p miR-98-5p miR-106b-5p miR-125-5p miR-133b miR-138-5p miR-181a-5p miR-181d-5p miR-203a-3p miR-298 miR-320a miR-342-3p miR-409-3p miR-432-5p	Anti-apoptotic effect	Joerger-Messerli et al. (2018)
Adipose-derived MSCs	Acute sciatic nerve injury	miR-195a-5p miR-184-3p miR-9-5p miR-137-3p miR-124-3p miR-132-3p	Neural differentiation	Roballo et al. (2019)
	Alzheimer’s disease	miRNA-22	Inhibit apoptosis, Anti-inflammatory effect	Zhai et al. (2021)
human Adipose derived MSCs	Peripheral nerve injury	miRNA-199b-5p	Modulate peripheral nerve-related cellular functions	Liu et al. (2022)
MSCs	Intracerebral hemorrhage	miR-133b	Neuroprotective role for anti-apoptotic effect	Shen et al. (2018)
PC12 cells and MSCs	Spinal cord injury	miR-21 miR-19b	Suppresses the apoptosis of neuron, increase cell differentiation	Xu et al. (2019)

### 4.2 Macrophage polarization role and peripheral nerve injury

**Figure 1:**
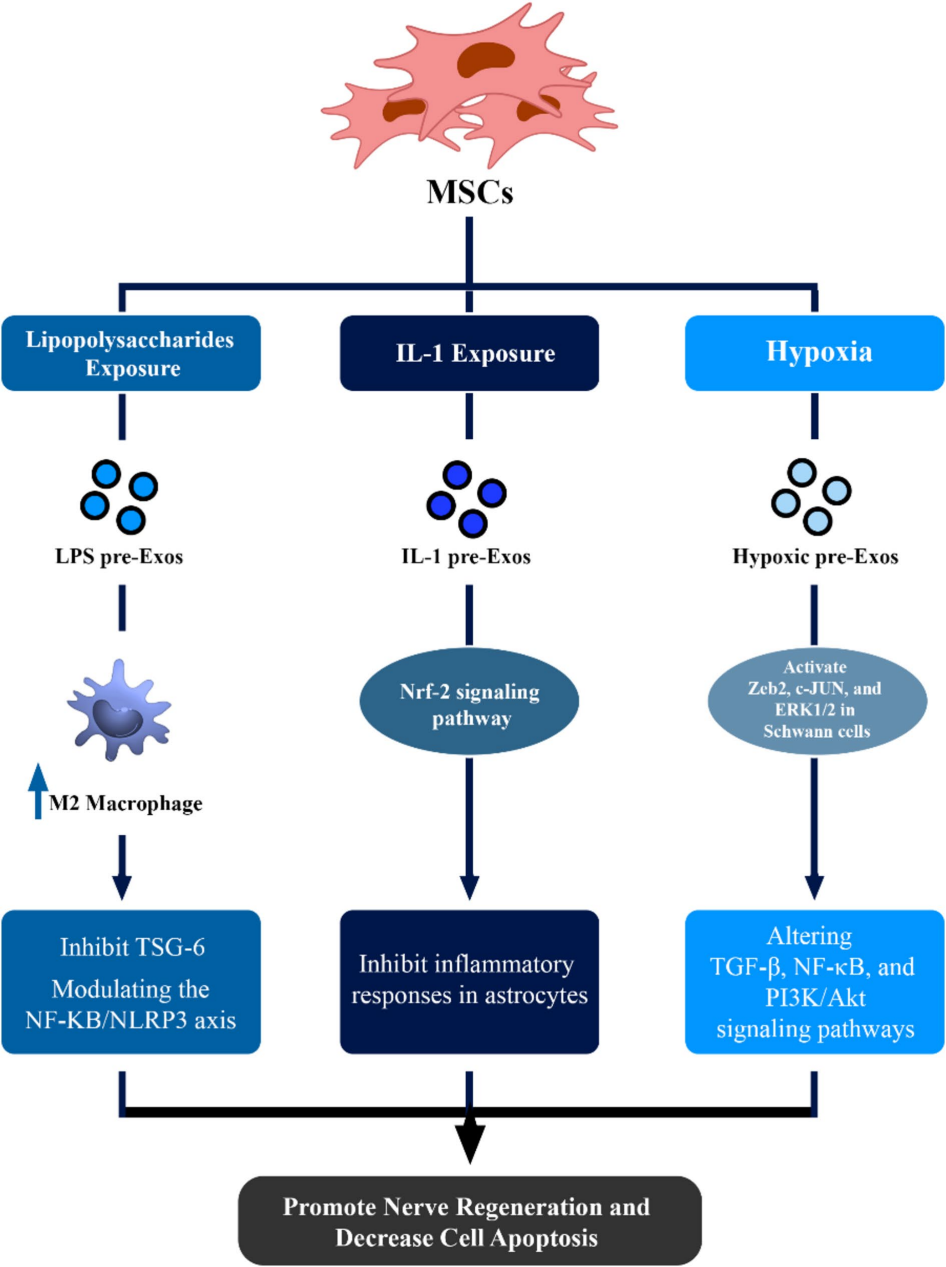
Therapeutic effects of mesenchymal stem cell (MSCs)-derived exosomes in neuronal regeneration and repair. This figure demonstrated that via alteration of condition media of MSCs, the potential of these exosomes to enhance regeneration of nerve cells can be increased via different pathways.

Exosomes derived from lipopolysaccharide (LPS) preconditioned MSCs (LPS pre-Exos) may facilitate the polarization of macrophages toward an M2 phenotype by inhibiting TNF-stimulated gene-6 (TSG-6). This subsequently modulates the NF-ΚB/NLRP3 axis (Li et al., 2022). This novel discovery presents a potential therapeutic strategy for peripheral nerve injury, demonstrating enhanced axon regrowth, remyelination, and improved M2 macrophage polarization upon local injection of LPS pre-Exos (Figure 1) (Li et al., 2022).

### 4.3 Utilizing IL-1 preconditioned MSC-EXOs

Treatment with IL-1 may greatly assist MSCs in the production of pro-trophic and anti-inflammatory compounds. IL-1 preconditioned MSC-EXOs can inhibit inflammatory responses in astrocytes and status epilepticus mice by acting via the Nrf-2 signaling pathway (Figure 1) (Liu et al., 2021).

### 4.4 Utilizing hypoxic preconditioned MSC-EXOs

Hypoxic conditions have been shown to play a critical role in enhancing the therapeutic impact of exosomes, particularly in hypoxia-related inflammatory conditions. Hypoxic conditioning of MSCs increases paracrine actions, altering key signaling pathways such as transforming growth factor *β* (TGF-β), NF-κB, and PI3K/Akt (Kang et al., 2019; Tirpe et al., 2019). Hypoxic preconditioning of umbilical cord MSC-EXOs can activate crucial components, including Zeb2, c-JUN, and ERK1/2 in Schwann cells, promoting nerve regeneration and myelin repair (Figure 1) (Zhu et al., 2022).

### 4.5 Neuroprotective properties of normoxic and hypoxic bone marrow MSC-EXOs

Moreover, Bone marrow MSC-EXOs and hypoxic Bone marrow MSC-EXOs can reveal strong neuroprotective properties against pyroptosis mediated by the NLRP3 inflammasome. Patients may benefit from using hypoxic bone marrow MSC-EXOs to slow the course of cerebral ischemia and hypoxia damage, as they have a more noticeable protective impact than normoxic bone marrow MSC-EXOs (Figure 1) (Kang et al., 2021).

### 4.6 Autologous MSC-EXOs

Researchers created a scaffold based on autologous plasma exosomes (AP-EXO), which were loaded with peptides that promote proliferation and target neurons. After inducing SCI in rats, 30 μg of this scaffold was injected intravenously and induced strong axon regeneration across the lesion core, reaching levels over 30-fold larger than naïve therapy. This helped to restore intraspinal networks and increase motor functional recovery. The AP-EXO-based tailored therapy, which combines safety and effectiveness, allows for functional recovery after SCI and has shown great promise for use in biomedical applications. Expanding the use of combinatory peptides and autologous exosomes produced from human plasma is beneficial in fostering regeneration and recovery during SCI therapy (Ran et al., 2023).

### 4.7 Non-autologous MSC-EXOs

For targeted therapeutics including CRISPR/Cas9 gene editing, exosomes provide a potentially effective delivery system. Using a subcutaneous tumor model, the *in vivo* treatment effectiveness of CRISPR/Cas9 packed MSC-EXOs was assessed by insertion of cultivated KPC689 cells in B6-albino mouse.

Mice were given a subcutaneous injection of one million KPC689 cells in the flank. 10^9^ CRISPR/Cas9-loaded exosomes (10^9^ exosomes, 10 μg plasmid DNA) were injected intratumorally. In syngeneic subcutaneous and orthotopic models of pancreatic cancer, exosomes equipped with CRISPR/Cas9 may target the mutant Kras^G12D^ oncogenic allele in pancreatic cancer cells to reduce proliferation and limit tumor development. This approach can be promising for neurological disorders as well (McAndrews et al., 2021).

## 5 Conclusion: future directions and implications for neurological therapeutics

### 5.1 Difficulties and solutions in using MSC-EXOs in therapeutic settings

There are several issues and difficulties with using MSC-EXOs in clinical settings. To comply with certain good manufacturing standards (GMP), translation of exosome-based clinical trials is necessary (Rezaie et al., 2022). Secondly, establishing uniformity in MSC culture and exosome separation techniques is required. A methodology for exosome separation, processing, identification, and functional dissection has been proposed earlier (Rai et al., 2021).

Thirdly, methods for effective delivery of exosomes to their intended locations are needed. To induce precise delivery, therapeutic exosomes can be genetically modified to express different targeting molecules. This can be done either directly, by adding covalent or non-covalent bonds to the exosomal surface, or indirectly, by genetically modifying the cells that produce exosomes (Choi et al., 2021).

Fourthly, recognizing the long-term safety and effectiveness of exosomes for nerve regeneration must be considered. Clinical research has shown that exosomes represent a promising therapeutic platform for several illnesses including Alzheimer’s disease, PD, SCI, PNI, and ALS. Although this exciting field is developing swiftly, more multidisciplinary research is required to address these little vesicles since we still have little understanding of the underlying mechanisms governing the many roles that exosomes play (Namini et al., 2023).

The comprehensive findings across studies underscore the potential of MSC-EXOs as a cell-free biological therapy for various neurological disorders. The ability of exosomes to modulate inflammatory responses, activate specific signaling pathways, and promote nerve regeneration positions them as promising candidates for future therapeutic interventions. Further research is warranted to elucidate the intricate mechanisms underlying exosome-mediated neuroprotection, offering new insights into neurological therapeutics and paving the way for innovative treatment strategies (Guo et al., 2019; Tirpe et al., 2019; Xu et al., 2022).

### 5.2 Future perspectives

As we move forward in the field of cellular regeneration, rejuvenation, and longevity of neuronal cells, further research is needed to uncover the specific miRNA cargo within exosomes and clarify their role in neural regeneration and protection. Standardizing isolation and characterization techniques will enhance the reproducibility of results. Moreover, it will be crucial for translational medicine to explore the potential of MSC-EXOs in clinical trials for a variety of neurological conditions. In conclusion, MSC-EXOs are at the forefront of innovative therapeutic strategies for neurological disorders. Their multifaceted impact on neuronal regeneration, modulation of inflammatory responses, and diverse applications highlight their potential importance in clinical settings. Continued research and clinical trials will pave the way for harnessing the full therapeutic potential of MSC-EXOs in addressing the complex challenges of neurological disorders.

## Glossary

MSCs: Mesenchymal stem cells
MSC-EXOs: Mesenchymal stem cells derived exosomes
EVs: extracellular vesicles
TGF-*β*: transforming growth factor β
TSG-6: TNF-stimulated gene-6
miRNA: micro RNA
Akt: protein kinase B
PI3K: phosphatidylinositol 3-kinase
ROS: reactive oxygen species
IVDD: intervertebral disc
Map2: microtubule-associated protein
CNPase: 2′,3′-cyclic-nucleotide 39-phosphodiesterase
Iba1: ionized calcium-binding adaptor molecule 1
GFAP: glial fibrillary acidic protein
GAP43: growth associated protein 43
NP: nucleus pulposus
mTOR: mammalian target of rapamycin
NLRP3: NOD-like receptor protein 3
MTX: methotrexate
IL-1: Interleukin-1beta
ALS: amyotrophic lateral sclerosis
SE: Status Epilepticus
PD: Parkinson’s disease
ONC: optic nerve crush
RGCs: retinal ganglion cells
AP-EXO: autologous plasma exosomes
